# Evolving Gender Gaps in Dental Anesthesiology: A Bibliometric Study of Anesthesia Progress

**DOI:** 10.7759/cureus.92606

**Published:** 2025-09-18

**Authors:** Atsuki Yamaguchi, Shota Tsukimoto, Hidetaka Kuroda, Aiji Sato-Boku, Ryo Wakita, Takuro Sanuki

**Affiliations:** 1 Department of Dental Anesthesiology, Kanagawa Dental University, Yokosuka, JPN; 2 Department of Dental Anesthesiology, Nagasaki University Hospital, Nagasaki, JPN; 3 Department of Anesthesiology, Aichi Gakuin University, Nagoya, JPN; 4 Department of Clinical Physiology, Nagasaki University School of Dentistry, Nagasaki, JPN

**Keywords:** academic publications, bibliometric analysis, dental anesthesiology, female first authors, gender gap

## Abstract

Closing the gender gap or achieving gender equality is a common goal in many fields. Achieving gender equality has positive impacts, such as increased diversity and specialized knowledge. A gender gap has been observed across various medical specialties, although recent trends suggest a gradual improvement in gender balance. Understanding the gender ratio in academic publications within this field can be considered essential for developing strategies to address gender disparities in dental anesthesiology academia. However, to date, no investigations have examined trends in authorship by gender in dental anesthesiology. We hypothesized that the gender gap in dental anesthesiology academic publications would also narrow. To test this hypothesis, we investigated the gender of the first author in *Anesthesia Progress*, the longest-running and most prolific journal in dental anesthesiology, over two periods. A total of 209 articles published between 2000 and 2003 and between 2020 and 2023 were collected from the Scopus database. For all collected articles, data including “first author,” “affiliation (country),” and “article type” were extracted. The first author was classified as female or male; the affiliation (country) as US, Japan, or other countries; and the type of article as “Review,” “Scientific report,” or “Case report.” Statistical analyses were performed using numerical data on the number of articles by gender and time period. Pearson’s chi-square test or Yates’ continuity-corrected chi-square test was applied with a significance level of 5%. The percentage of articles with female first authors increased significantly, from 19.2% (10/52) in the 2000s to 34.0% (49/144) in the 2020s (P = 0.046). By country, the percentage of female first authors decreased in the US but increased in Japan and other countries. Notably, in Japan, the proportion of female first authors rose significantly from 1.9% (1/52) in the 2000s to 22.2% (32/144) in the 2020s (P = 0.002). Regarding the types of articles submitted by female first authors, the number of “Scientific report” articles markedly decreased in the US, whereas the number of “Case report” articles significantly increased in Japan. The investigation revealed that the gender gap in dental anesthesiology has narrowed in terms of total representation; however, the extent of this gap differs from country to country. Regular statistical bibliometric analyses could provide more insights into closing the gender gap.

## Introduction

Achieving gender equality was listed as one of 17 international goals for sustainable development adopted by the United Nations General Assembly in 2015 [1]. Gender equality is not only a fundamental human right but also a necessary foundation for peace and prosperity. Women have often been undervalued in society, and enabling their participation in decision-making processes will promote sustainability and benefit both society and humanity as a whole [2]. Therefore, closing this gender gap has become a common goal in various fields.

In the medical field, the percentage of female doctors and dentists in each country has been increasing in recent years [3-5]. However, there is a significant gender gap in academic medicine [5,6]. This may have a negative impact on individuals and society, such as restraining the growth of individual careers and hindering advances in professional knowledge in the field [4,7].

Considering that the number of articles published in medical journals is an important measure of academic productivity and provides insights into academic activity [5], bibliometric analysis has been conducted in various specialties (nephrology, arrhythmia research (cardiology), gastroenterology, surgery, and psychiatry) [8-12].

Dental anesthesiology is a specialized field that focuses on the management of anesthesia during complex dental procedures. Over the past decades, it has developed into a well-recognized academic discipline, particularly in developed countries [13,14]. Despite these advancements, no comprehensive studies have been conducted to investigate gender-specific authorship trends in the dental anesthesiology literature. Understanding the gender ratio in academic publications within this field can be considered essential for developing strategies to address gender disparities in dental anesthesiology academia.

*Anesthesia Progress*, the journal published by the American Dental Society of Anesthesiology and first published in 1966, is the longest-running and most prolific journal dedicated to dental anesthesiology [15]. Given its historical significance and consistent publication records, it is an ideal source for examining longitudinal trends in gender authorship. We hypothesized that the gender gap among first authors in dental anesthesiology has narrowed over time. To test this hypothesis, we examined how gender representation among the first authors in *Anesthesia Progress* shifted over time.

## Materials and methods

The journal used for the analysis was *Anesthesia Progress*, the longest-running and most prolific journal in the field of dental anesthesiology. Given that this was a retrospective study using a database, the requirement of an ethics review at our facility was waived. Data for these articles were obtained from the Scopus database on January 9, 2024.

We collected data on all articles published in *Anesthesia Progress* using the electronic database Scopus. The search was performed by entering “*Anesthesia Progress*” in the “Source Title” field. Only documents categorized as “Article” or “Review” were included, and the data were divided into two time periods: 2000-2003 and 2020-2023. The latter period was selected because data reflecting the most recent female representation were compiled in 2024, covering the years 2020-2023. For comparison, the first four years of the 2000s were also analyzed.

We extracted data including “first author,” “affiliation (country),” and “article type” from the database of collected article data. The first author was classified as either female or male. The gender of the first author’s journal was confirmed by a web search, not by a program that automatically identified the gender. To determine the authors’ gender, we referred to their official profiles on their affiliated institutions’ websites and the Japanese Ministry of Health, Labour and Welfare’s physician and dentist directory. When gender information was available, we classified authors accordingly. If no explicit information was found, we inferred gender based on their names and photos, using common knowledge and context.

Affiliation (country) was classified as US, Japan, or other countries. Articles without authors, such as comments, editorials, reprints of previous articles, question articles, or error corrections, were excluded from the survey because they were not considered to be research articles submitted for publication. The remaining articles were categorized into three types: “Review”, “Scientific report”, and “Case report.” The type of each article was classified based on the description provided in the article, if available. If no such description was found, the classification was made at the authors’ discretion.

Statistical analyses were performed using numerical data on the number of articles by gender and time period, with a significance level of 5%. Pearson’s chi-square test or Yates’ continuity-corrected chi-square test was applied as appropriate, depending on the expected cell counts. Specifically, Yates’ continuity correction was applied when any expected frequency in a 2 × 2 contingency table was less than 10. All statistical analyses were performed using GraphPad Prism 7.05 (GraphPad Software, La Jolla, CA, US).

## Results

**Figure 1:**
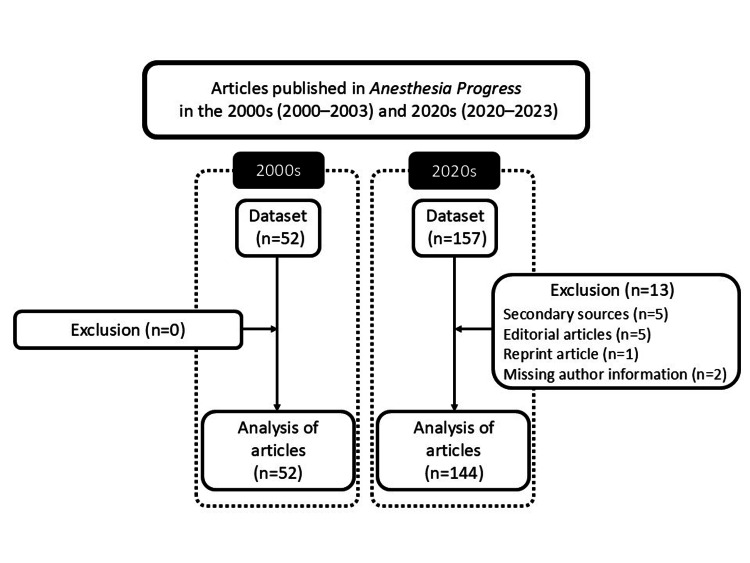
Study design A total of 209 articles were published in *Anesthesia Progress*: 52 in the 2000s and 157 in the 2020s. 2000s: the four-year period from 2000 to 2003; 2020s: the four-year period from 2020 to 2023

**Figure 2:**
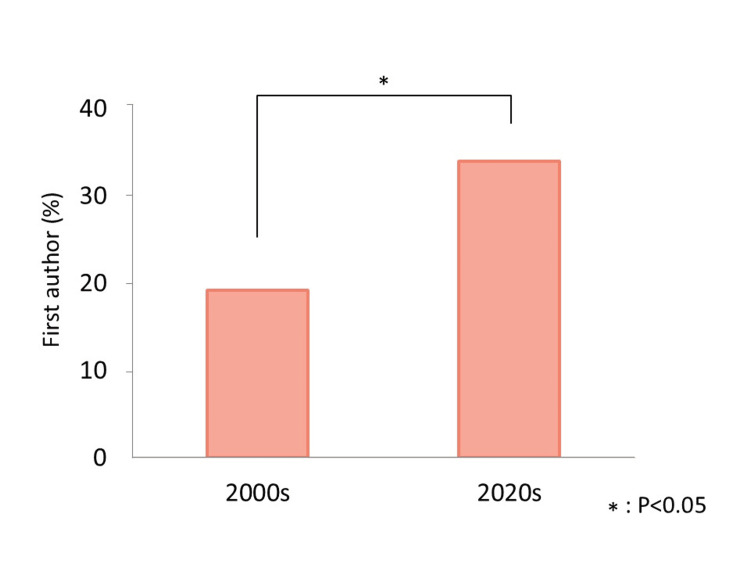
Changes in the percentage of female first authors in each group The percentage of female first authors is shown as a bar graph for each group. From the 2000s to the 2020s, this percentage increased significantly from 19.2% to 34.0% (P = 0.046). 2000s: the four-year period from 2000 to 2003; 2020s: the four-year period from 2020 to 2023

A total of 209 articles were published in *Anesthesia Progress*: 52 from 2000 to 2003 (2000s) and 157 from 2020 to 2023 (2020s). After excluding studies that met the exclusion criteria, 52 articles from the 2000s and 144 from the 2020s were analyzed (Figure 1, Table 1, Table 2). The percentage of articles with a female first author increased significantly from 19.2% (10/52) in the 2000s to 34.0% (49/144) in the 2020s (Figure 2, Table 3; χ² = 3.976, P = 0.046).

**Table 1:** List of articles by female first authors in the 2000s

No.	First author	Article title	Country of affiliation	Article type	Year of publication
1	Ridenour S	Anesthetic efficacy of a combination of hyaluronidase and lidocaine with epinephrine in inferior alveolar nerve blocks	US	Scientific report	2001
2	Kaakko T	Recruiting phobic research subjects: effectiveness and cost	US	Scientific report	2001
3	Lee JY	A cost analysis of treating pediatric dental patients using general anesthesia versus conscious sedation	US	Scientific report	2001
4	Noguchi I	Pain relief by stellate ganglion block in a case with trigeminal neuralgia caused by a cerebellopontine angle tumor	Japan	Case report	2002
5	Bui T	A comparison study between ketamine and ketamine-promethazine combination for oral sedation in pediatric dental patients	US	Scientific report	2002
6	Stecker SS	Should a mucoadhesive patch (DentiPatch) be used for gingival anesthesia in children?	US	Scientific report	2002
7	Leopold A	Pharmacokinetics of lidocaine delivered from a transmucosal patch in children	US	Scientific report	2002
8	Gallatin J	A comparison of injection pain and postoperative pain of two intraosseous anesthetic techniques	US	Scientific report	2003
9	Tófoli GR	Comparison of effectiveness of 4% articaine associated with 1: 100,000 or 1: 200,000 epinephrine in inferior alveolar nerve block	Brazil	Scientific report	2003
10	White H	Parental evaluation of quality of life measures following pediatric dental treatment using general anesthesia	US	Scientific report	2003

**Table 2:** List of articles by female first authors in the 2020s

No.	First author	Article title	Country of affiliation	Article type	Year of publication
1	Ikeda N	Combined use of a gum elastic bougie and video laryngoscopy for intubating a patient with an unexpected laryngeal papilloma	Japan	Case report	2020
2	Park L	Comparing the efficacy of a compound topical anesthetic versus benzocaine: a pilot study	US	Scientific report	2020
3	Dowdy RA	Medical management of epiglottitis	US	Case report	2020
4	Kaushik M	Comparing the efficacy of twin mix and lidocaine for inferior alveolar nerve blocks in patients with symptomatic irreversible pulpitis	India	Scientific report	2020
5	Koyanagi Y	A case of successful tracheal tube exchange with McGrath MAC for tube damage during oral surgery	Japan	Case report	2020
6	Hayashi R	Pulmonary aspiration during induction of general anesthesia	Japan	Case report	2020
7	Sakamizu A	Anesthetic management for an adult with glycogen storage disease type 0	Japan	Case report	2020
8	Yamashita K	General anesthesia during lip repair and palatoplasty after Glenn surgery	Japan	Case report	2020
9	Chogyoji Y	Impact of subglottic saline irrigation on reducing bacterial contamination for oral surgery patients	Japan	Scientific report	2020
10	Tsutsui Y	Adding dexmedetomidine to articaine increases the latency of thermal antinociception in rats	Japan	Scientific report	2020
11	Katagiri N	Postoperative pain management in patients with ulcerative colitis	Japan	Case report	2020
12	Shin J	Anesthetic management of the pregnant patient: part 2	US	Review: continuing education	2021
13	Maekawa M	Anesthetic management of a patient with ring 18 syndrome	Japan	Case report	2021
14	Takaishi K	A retrospective case series of anesthetic patients with epiglottic cysts	Japan	Case report	2021
15	Lee S	Kawasaki disease and general anesthesia for dental treatment: a case report	US	Case report	2021
16	Takaishi K	Management of a patient with tracheal stenosis after previous tracheotomy	Japan	Case report	2021
17	Lipp K	Effect of intrapapillary local anesthetic on postoperative pain following dental treatment under general anesthesia in pediatric patients	US	Scientific report	2021
18	Hashimoto M	A case of childhood-onset Basedow-Graves’ disease diagnosed as a result of the fourth time of general anesthesia for oral and maxillofacial surgery	Japan	Case report	2021
19	Arai Y	A case of nasal mucosa cautery with reintubation under pharyngeal suction for massive epistaxis after extubation	Japan	Case report	2021
20	Fazeli A	Cardiovascular safety and hemostatic efficacy of topical epinephrine in children receiving zirconia crowns	US	Scientific report	2021
21	Wong M	Ambulatory anesthesia for a case of idiopathic bronchiolitis obliterans	Canada	Case report	2021
22	Shibuya M	Anesthetic management of a patient with citrullinemia type I during dental treatment	Japan	Case report	2021
23	Yasuda A	Anesthetic management of a juvenile hyaline fibromatosis patient with trismus and cervical movement limitation	Japan	Case report	2021
24	Shibuya M	Cardiovascular considerations in anesthetic management for a patient with antiphospholipid syndrome and decreased cardiac function: a case study	Japan	Case report	2021
25	Dowdy RA	Cardiac arrest upon induction of general anesthesia	US	Case report	2021
26	Shin J	Anesthetic management of the pregnant patient: part 1	US	Review	2021
27	Oda W	Clinical use of preformed Microcuff^®^ pediatric endotracheal tubes in Japan	Japan	Scientific report	2021
28	Taharabaru S	Difficult airway management in a patient with Nicolaides-Baraitser syndrome who had a small jaw and limited mouth opening	Japan	Case report	2021
29	Eriguchi A	Effects of remifentanil on cardiovascular stimulation caused by local anesthetic with epinephrine: a power spectral analysis	Japan	Scientific report	2021
30	Takagi S	Methemoglobinemia induced by prilocaine in a child with Noonan syndrome	Japan	Case report	2022
31	Usami N	A case of wide QRS tachycardia after the local administration of epinephrine to reduce bleeding during general anesthesia	Japan	Case report	2022
32	Teshima R	Anesthesia management of a patient with familial cold autoinflammatory syndrome: a case report	Japan	Case report	2022
33	Sasaki H	Severe bleeding during orthognathic surgery for a Noonan syndrome patient	Japan	Case report	2022
34	Wong M	Reversal agents in sedation and anesthesia practice for dentistry	Canada	Review	2022
35	Kuroda I	General anesthesia for a dissociative identity disorder patient with 20 personalities: a case report	Japan	Case report	2022
36	Ishikawa E	Cross-sectional study of PONV risk factors for oral surgery after intubated general anesthesia with total intravenous anesthesia	Japan	Scientific report	2022
37	Shinoda M	Optimal timing of intravenous acetaminophen administration for postoperative analgesia	Japan	Scientific report	2022
38	Waters CM	ACE-inhibitor or ARB-induced refractory hypotension treated with vasopressin in patients undergoing general anesthesia for dentistry: two case reports	US	Case report	2022
39	Sasaki S	Successful tracheal intubation with airway scope after failure with McGrath	Japan	Case report	2023
40	Compton P	Preoperative and postoperative hyperalgesia in dental patients on chronic opioid therapy: a pilot study	US	Scientific report	2023
41	Smith T	Management of an ingested foreign body in a COVID-positive patient	US	Case report	2023
42	Sumphaongern T	Sudden cardiac arrest in a dental patient awaiting examination	Thailand	Case report	2023
43	Woo A	Success of pulpal anesthesia following buccal infiltration of the maxillary first molar with 1.8 mL and 3.6 mL of 4% articaine with 1:100,000 epinephrine: a prospective, randomized crossover study	US	Scientific report	2023
44	Nishikawa M	Pronounced QT prolongation during general anesthesia in a child with left ventricular noncompaction cardiomyopathy: a case report	Japan	Case report	2023
45	Fujii-Abe K	A case of anterior arytenoid cartilage dislocation during nasal tracheal intubation using an indirect video laryngoscope	Japan	Case report	2023
46	Takeda S	Anesthetic management of a patient with spinocerebellar ataxia type 1	Japan	Case report	2023
47	Shiraishi K	Lidocaine tape application for 3 hours prevents vasovagal syncope during venipuncture: a case series	Japan	Case report	2023
48	Rafla RR	Comparison of oropharyngeal oxygen pooling and suctioning during intubated and nonintubated dental office-based anesthesia	US	Scientific report	2023
49	Nishioka Y	Anesthetic management using remimazolam in a hemodialysis patient	Japan	Case report	2023

**Table 3:** Female first authors in the 2000s (2000-2003) and 2020s (2020-2023): distribution by countries and article types This table shows the proportion and number of female first authors among all articles published in the 2000s (2000-2003) and the 2020s (2020-2023). For each period, the data are presented overall (combined sexes) and stratified by the affiliation country of the first author (US, Japan, and other countries) and by article type (review, scientific report, and case report). “Other countries” include Brazil (one entry in the 2000s) and Canada (two entries), India (one entry), and Thailand (one entry) in the 2020s. P-values were calculated using chi-square tests based on the number of articles according to gender (female vs. male) and periods (2000s vs. 2020s).

	2000s (n = 52)		2020s (n = 144)		Chi-value	P-value (2000s vs. 2020s)
Overall	19.20%	(10/52)	34.00%	(49/144)	3.976	0.046
Review	0%	(0/52)	2.10%	(3/144)	0.152	0.697
Scientific report	17.30%	(9/52)	9.00%	(13/144)	1.863	0.172
Case report	1.90%	(1/52)	22.90%	(33/144)	10.325	0.001
US	15.40%	(8/52)	9.00%	(13/144)	1.018	0.313
Review	0%	(0/52)	1.40%	(2/144)	0.002	0.961
Scientific report	15.40%	(8/52)	4.20%	(6/144)	5.656	0.017
Case report	0%	(0/52)	3.40%	(5/144)	0.719	0.396
Japan	1.90%	(1/52)	22.20%	(32/144)	9.84	0.002
Review	0%	(0/52)	0%	(0/144)	-	-
Scientific report	0%	(0/52)	4.20%	(6/144)	1.052	0.305
Case report	1.90%	(1/52)	18.00%	(26/144)	7.068	0.008
Others	1.90%	(1/52)	2.80%	(4/144)	0.032	0.859
Review	0%	(0/52)	0.70%	(1/144)	0.284	0.594
Scientific report	1.90%	(1/52)	0.70%	(1/144)	0.002	0.961
Case report	0%	(0/52)	1.40%	(2/144)	0.002	0.967

The percentages of female first authors in the US, Japan, and other countries were 15.4% (8/52), 1.9% (1/52), and 1.9% (1/52) in the 2000s and 9.0% (13/144), 22.2% (32/144), and 2.8% (4/144) in the 2020s, respectively. The percentage of female first authors in Japan increased significantly from 1.9% (1/52) in the 2000s to 22.2% (32/144) in the 2020s (Table 3; χ² = 9.84, P = 0.002).

On examining the types of articles submitted by female first authors by country in the 2000s and 2020s, in the US, the percentage of “Scientific report” articles decreased from 15.4% (8/52) to 4.2% (6/144), whereas the percentages of “Review” and “Case report” articles increased over time from 0% (0/52) to 1.4% (2/144) and from 0% (0/52) to 3.4% (5/144), respectively. In other countries, the percentage of “Scientific report” articles decreased from 1.9% (1/52) to 0.7% (1/144), whereas the percentages of “Review” and “Case report” articles increased over time from 0% (0/52) to 0.7% (1/144) and from 0% (0/52) to 1.4% (2/144), respectively, similar to the trend seen in the US. In Japan, the percentages of “Scientific report” and “Case report” increased over time from 0% (0/52) to 4.2% (6/144) and from 1.9% (1/52) to 18.0% (26/144), respectively. The percentage of “Review” articles remained unchanged at 0% (0/52, 0/144) (Table 3).

## Discussion

A survey using an electronic database, known as a bibliometric analysis, may provide novel insights into the fairness and diversity in scientific research [16]. Currently, three journals are published by academic societies specializing in dental anesthesiology: *Anesthesia Progress*, *Journal of Dental Anesthesia and Pain Medicine*, and *Journal of the Japanese Dental Society of Anesthesiology *[15]. We focused on *Anesthesia Progress*, one of the three journals specializing in dental anesthesiology, which has the largest number of articles in the Scopus database and the oldest publication history. By examining this journal, we speculated that it is possible to evaluate the shifting trends in dental anesthesiology. Given the growing momentum of international efforts to improve the status of women over the past two decades [17], this study compared two time periods, the 2000s and the 2020s, and obtained the following results.

First, the overall percentage of female first authors significantly increased. Second, the percentage of female first authors decreased in the US while it increased in Japan and other countries, with a particularly significant increase in Japan. Third, regarding the types of articles submitted by female first authors, the number of “Scientific report” articles markedly decreased in the US, whereas the number of “Case report” articles significantly increased in Japan. These results generally support the hypothesis that the gender gap among first authors in dental anesthesiology has narrowed over time, although this trend is not consistently observed across all categories.

The percentage of female first authors in the field of dental anesthesiology is increasing, suggesting that the gender gap in dental anesthesiology is being addressed. This result is consistent with previous findings on academic publications in multiple specialized disciplines [8-12].

In general, the gender gap is considered to have widened for the following reasons: low access to research funding, lack of investment in research-related infrastructure [8,11,18-21], prejudice regarding the reliability of research activities [9,10], low number of female supervisors [5,19,22,23], the significant amount of time required for household chores and childcare [23-25], and cultural differences between countries. For example, as women in the US advance in their academic careers, the number of female researchers has decreased [5,10]. Consequently, there were fewer female professors and research leaders [5,6,9,12,22].

The above is considered to be one of the reasons why the number of female first authors of “Scientific report” articles is not increasing in the US. In contrast, in Japan, the number of female dentist anesthesiologists belonging to academic organizations (the Japanese Dental Society of Anesthesiology) is steadily increasing. Furthermore, since publication in *Anesthesia Progress* is a prerequisite for taking the qualification examination administered by this organization, it is possible that this has contributed to an increase in the number of female first authors in Japan. Nevertheless, female first authors remain underrepresented in “Scientific report” articles, which are typically associated with high academic innovation.

Various initiatives are underway to reduce this gender gap in academia. It is necessary to facilitate a supportive environment for women in research, minimize gender bias, improve the work-life balance, and promote the introduction of flexible career systems [9,19,26,27]. However, given that the content and progress of these efforts vary across academic fields and countries/regions, the gender gap must be regularly evaluated, and changes must be investigated.

This study has several limitations that need to be addressed. First, the number of articles examined may be small as a sample size. Second, the data obtained were exclusively from *Anesthesia Progress*, which does not include all studies in the field of dental anesthesiology and may also have been affected by a number of factors, such as the ease of submission to *Anesthesia Progress* and the existence of other national and international journals, which may have introduced bias. This suggests that the results may not be generalizable to the entire field of dental anesthesiology. Third, only accepted articles were included in the survey; articles that were not accepted for any reason were excluded. Fourth, because we focused solely on the first author of the publication, we were unable to include information on other authors. Fifth, the interpretation of the results was limited by the small sample sizes for countries other than Japan and the US, where the first authors were affiliated. Therefore, classifying affiliations into three categories (the US, Japan, and other countries) may have oversimplified the geographical diversity. Sixth, the influence of cultural backgrounds and institutional differences across countries on publication behavior has not yet been thoroughly investigated. Finally, the accuracy of the data cannot be guaranteed owing to the use of online searches.

## Conclusions

The gender gap in dental anesthesiology has narrowed in terms of total representation; however, the extent of this gap varies across countries. Closing the gender gap in academia will lead to higher-quality research and greater diversity.

Regular statistical bibliometric analysis of gender gaps can be used as a means of formulating specific measures to improve the research environment. Extending such analysis to other journals in dental anesthesiology, particularly by including data on the gender of last authors, may provide a more comprehensive understanding of gender disparities in this field.

## References

[1] Leal Filho W, Kovaleva M, Tsani S (2022). Promoting gender equality across the sustainable development goals. Environ Dev Sustain.

[2] (2024). Sustainable Development Goals. https://www.un.org/sustainabledevelopment/sustainable-development-goals/.

[3] Ministry of Health L and W (2024). Ministry of Health L and W: Summary of Statistics on Physicians, Dentists, and Pharmacists in 2020 [in Japanese]. https://www.mhlw.go.jp/toukei/saikin/hw/ishi/20/index.html.

[4] Penny M, Jeffries R, Grant J, Davies SC (2014). Women and academic medicine: a review of the evidence on female representation. J R Soc Med.

[5] Jagsi R, Guancial EA, Worobey CC (2006). The “gender gap” in authorship of academic medical literature — a 35-year perspective. N Engl J Med.

[6] Bickel J, Wara D, Atkinson BF (2002). Increasing women's leadership in academic medicine: report of the AAMC Project Implementation Committee. Acad Med.

[7] Abraham J, Panchal K, Varshney L, Lakshmi Narayan K, Rahman S (2023). Gender disparities in first authorship in publications related to attention deficit hyperkinetic disorder (ADHD) and artificial intelligence (AI). Cureus.

[8] Abraham RR, Adisa O, Owen ME, Iqbal F, Sulaiman K (2023). Evaluation of gender trends in first authorship in nephrology publications in four major US journals in the last decade. J Nephrol.

[9] Djahanshahi N, Seelamanthula S, Shubhangi F, Jagarlamudi NS, Dhawan A, Spandana VV (2023). Gender trends in first authorship of academic publications related to Wolff-Parkinson-White syndrome. Cureus.

[10] Foley C, Harewood G, Benz E, Higgins L, Gibbons E, Kelly S, Cheriyan D (2022). Gender equality in academic gastroenterology: a review of gastroenterology literature over four decades. Ir J Med Sci.

[11] Motter SB, Brandão GR, Iaroseski J (2021). Gender-related trends in publication authorship: a 10-Year analysis of a Brazilian surgical journal. Cureus.

[12] Hart KL, Frangou S, Perlis RH (2019). Gender trends in authorship in psychiatry journals from 2008 to 2018. Biol Psychiatry.

[13] Weaver JM (2019). The history of the specialty of dental anesthesiology. Anesth Prog.

[14] Sanuki T, Miyawaki T, Iijima T (2023). A survey on the safety of anesthesia management provided by dental anesthesiologists in Japan: a 5-year survey by the Japanese Dental Society of Anesthesiology. Clin Oral Investig.

[15] Sanuki T, Tsukimoto S, Kuroda H, Kido K (2024). Impact factor for journals specializing in dental anesthesiology. Anesth Prog.

[16] Donthu N, Kumar S, Mukherjee D, Pandey N, Lim WM (2021). How to conduct a bibliometric analysis: an overview and guidelines. J Bus Res.

[17] Tyer-Viola LA, Cesario SK (2010). Addressing poverty, education, and gender equality to improve the health of women worldwide. J Obstet Gynecol Neonatal Nurs.

[18] Mahajan A, K V, Dikshit N, Sandhu JK, Pallempati LL, Olivieri L (2024). Gender representation in academic publications of Tourette syndrome research: an analysis of authorship trends. Cureus.

[19] Bates C, Gordon L, Travis E (2016). Striving for gender equity in academic medicine careers: a call to action. Acad Med.

[20] Cain JM, Schulkin J, Parisi V, Power ML, Holzman GB, Williams S (2001). Effects of perceptions and mentorship on pursuing a career in academic medicine in obstetrics and gynecology. Acad Med.

[21] Benz EJ, Clayton CP, Costa ST (1998). Increasing academic internal medicine’s investment in female faculty. Am J Med.

[22] Yedidia MJ, Bickel J (2001). Why aren't there more women leaders in academic medicine? the views of clinical department chairs. Acad Med.

[23] Vijayakumar V, Babu HF, Karki A, Tyagi R, Macapia M, Zapata KM, Dogiparthi S (2023). Gender disparity of first authors in review article publications related to schizophrenia. Cureus.

[24] (2012). Nature’s sexism. Nature.

[25] Carr PL, Ash AS, Friedman RH (1998). Relation of family responsibilities and gender to the productivity and career satisfaction of medical faculty. Ann Intern Med.

[26] Westring A, McDonald JM, Carr P, Grisso JA (2016). An integrated framework for gender equity in academic medicine. Acad Med.

[27] Varkey P, Jatoi A, Williams A (2012). The positive impact of a facilitated peer mentoring program on academic skills of women faculty. BMC Med Educ.

